# Association between selective digestive decontamination and decreased rate of acquired candidemia in mechanically ventilated ICU patients: a multicenter nationwide study

**DOI:** 10.1186/s13054-023-04775-1

**Published:** 2023-12-16

**Authors:** Florian Reizine, Nicolas Massart, Vincent Joussellin, Anaïs Machut, Charles-Hervé Vacheron, Anne Savey, Arnaud Friggeri, Alain Lepape, the REAREZO study group, Serge Alfandari, Alexandra Allaire, Antonio Alvarez, Ammenouche Nacim, Laurent Argaus, Gérard Audibert, Caroline Aurel, Odile Bajolet, Frédéric Barbut, Genevieve Barjon, Patricia Baune, Sébastien Beague, Bassam Beilouny, Nicolas Bele, Nicolas Belin, Cécile Bernerd, Yasmina Berrouane, Aziz Berrouba, Julie Bertrand, Claire Bianchi, Sandrine Biangoma, Fabienne Birot Jaulin, Severine Bonjean, Stéphanie Bordes-Couecou, Abdenour Bouhara, Philippe Bouillard, Céline Bourigault, Sylvie Bourzeix, Sébastien Boutreux, Hanene Bouzidi, Julie Brochart, Stéphanie Bulyez, Marie Callanquin, Nathalie Canu, Matthieu Capron, Daniel Carbognani, Vincent Castelain, Vincent Catanese, Isabelle Cattaneo, Vanessa Chartier, Guillaume Chassaing, Robert Chausset, Mélanie Chauvet, Fabrice Chopin, Catherine Chubilleau, Céline Clayer, Agnès Cohen, Sylvie Comparot, Philippe Corne, Marie-Elisabeth Cornesse, Gaelle Corno, Esther Cortes, Patricia Courouble, Christian Crombe, Véronique Curnier, Monzer Dabbachi, Cédric Dananché, Abla Daroukh, Damien Dassant, Martine Daumas, Aurélien Daurat, Dominique Deffarges, Fanny Delanghe, Olivier Delastre, Joel Delhomme, Jean Paul Délias, Martine Delorme, Fabienne Derramond, Frédérique Diaw, Isabelle Dijols, Kamel Djedaini, Loic Dopeux, Sophie Duhoo, Thierry Dulac, Clarisse Dupin, Laurène Dupont, Michel Durand, Isabelle Durand Joly, Jean-Yves Dusseau, Pierre Yves Egreteau, Carole Eldin, Florence Espinasse, Eric Farfour, Abdelhamid Fatah, Yannick Fedun, Luis Ferreira, Pierre Fillatre, Toufic Finge, Véronique Fleurial, Arnaud Florentin, Agnès Fribourg, Severine Gallais-Hoff, Claude Galland, Richard Galliot, Sylvain Garnier, Gaelle Gasan, Julien Gaubert-Duclos, Valérie Gauzere, Thomas Geffriaud, Isabelle Geneau, Hughes Georges, Solweig Gerbier Colomban, Christophe Giacardi, Sebastien Gibot, Audrey Glanard, Marion Gleize, Marieline Goret, Michele Gourgues, Delphine Grau, Béatrice Grisi, Clotilde Groleau, Liliane Grolier-Bois, Catherine Guignabert, Fethi Hadj-Slimane, Emmanuelle Hammad, Catherine Haond, Marie Hélène Hausermann, Francoise Hayo, Christophe Henry, Alexandre Herbland, Julien Huntzinger, Hervé Hyvernat, Alexandre Jean, Boris Jolibois, Sylvie Joron, Gauthier Julien, Jean Kempf, Lyes Knani, Béatrice La Combe, Marie Labruyere, Sandrine Lacroix, Bruno Lafon, Katia Lamant, Peggy Larroudé, Anne Launoy, Bernadette Laurent, Thierry Lavigne, Christine Lawrence, Quoc Vien Le, Muriel Le Coq, Anne-Sophie Le Floch, Fanny Le Fall, Brigitte Le Tallec, Lucie Lecoutre, Stanislas Ledochowski, Rusel Leon, Claire Lepouse, Thomas Lescot, Mélanie Levrard, Marie Laure Lier, Anne Lignereux, Benjamin Louart, Claire Maheu, Aurélie Maindron, Francois Mallard, Marie Reine Mallaret, Bernard Mankikian, Christiane Manzon, Philippe Mardrus, Jacques Mariot, Audry Martin, Emmanuelle Martin, Maelle Martin, Pascale Martres, Virginie Maxime, Olivier Meunier, Ella-Pauline Meyer, Ferhat Meziani, Sébastien Moschietto, Céline Muller, Elodie Munier-Marion, Caroline Neulier, François Nicolas, Jacques-Yves Nizou, Christine Palitta, Michel Pascal, Olivier Passouant, René-Gilles Patrigeon, Frédérique Pavillard, Sabine Peghaire, Christophe Perdrix, Jean-Sebastien Petit, Judith Pibre, Walter Picard, Sylvie Picault, Santiago Picos Gil, Jérôme Pillot, Patrick Pina, Rémi Plattier, Laurent Poiron, Christian Pommier, Gaël Pradel, Cristian Prelipcean, Paul-Simon Pugliesi, Vincent Quenee, Olga Raposo, Eve Remy, Sabine Reynaud Deforges, Jean-Christophe Richard, Sylvie Ricome, Thomas Rimmele, Adrien Robine, Anne-Claude Roche, Laetitia Rohr, Gwenaël Rolland-Jacob, Adrien Roques, Catherine Rougier, Jérôme Roustan, Mélanie Saint-Leger, Faouzi Saliba, Dominique Sechaud, Amine Si-Ali, Catherine Simac, Georges Simon, Michel Sirodot, Vincent Stoeckel, Philippe Tagawa, Marine Tasle, Fabrice Thiollière, Benoit Thiphagne, Aurélie Thomas-Hervieu, François Tinturier, Alexandre Tonnelier, Alexandre Toro, David Tranvan, Dominique Trivier, Gilles Troché, Rémi Trusson, Lionel Ursulet, Marie Laure Valdeyron, Catherine Vallet, Vanessa Van Rossem, Laurence Vasse, Myriam Venelle, Christine Venot, Antoine Vieillard-Baron, Jean-François Vincent, Michel Vitris, Hussein Yassine, Lassane Zanre, Cecile Zylberfajn

**Affiliations:** 1 Service de Réanimation Polyvalente, Centre Hospitalier de Vannes, 56000 Vannes, France; 2 Service de Réanimation Polyvalente, Centre Hospitalier de Saint Brieuc, 22000 Saint-Brieuc, France; 3 REA-REZO Infections et Antibiorésistance en Réanimation, Hôpital Henry Gabrielle, 69230 Saint-Genis-Laval, France; 4 Département d’Anesthésie Médecine Intensive Réanimation, Centre Hospitalier Lyon Sud, Hospices Civils de Lyon, 165 Chemin du Grand Revoyet, 69310 Pierre-Bénite, France; 5 Centre International de Recherche en Infectiologie, Institut National de La Santé et de la Recherche Médicale U1111, CNRS Unité Mixte de Recherche 5308, École Nationale Supérieure de Lyon, Université Claude Bernard Lyon 1, PHE3ID Villeurbanne, France

**Keywords:** Candidemia, Selective digestive decontamination, ICU

## Abstract

**Background:**

Candidemia is a high-risk complication among intensive care unit (ICU) patients. While selective digestive decontamination (SDD) has been shown to be effective in preventing ICU-acquired bacterial secondary infection, its effects on ICU-acquired candidemia (ICAC) remain poorly explored. Therefore, we sought to assess the effects of SDD on ICAC.

**Method:**

Using the REA-REZO network, we included adult patients receiving mechanical ventilation for at least 48 h from January 2017 to January 2023. Non-parsimonious propensity score matching with a 1:1 ratio was performed to investigate the association between SDD and the rate of ICAC.

**Results:**

A total of 94 437 patients receiving at least 48 h of mechanical ventilation were included throughout the study period. Of those, 3 001 were treated with SDD and 651 patients developed ICAC. The propensity score matching included 2 931 patients in the SDD group and in the standard care group. In the matched cohort analysis as well as in the overall population, the rate of ICAC was lower in patients receiving SDD (0.8% versus 0.3%; *p* = 0.012 and 0.7% versus 0.3%; *p* = 0.006, respectively). Patients with ICAC had higher mortality rate (48.4% versus 29.8%; *p* < 0.001). Finally, mortality rates as well as ICU length of stay in the matched populations did not differ according to SDD (31.0% versus 31.1%; *p* = 0.910 and 9 days [5–18] versus 9 days [5–17]; *p* = 0.513, respectively).

**Conclusion:**

In this study with a low prevalence of ICAC, SDD was associated with a lower rate of ICAC that did not translate to higher survival.

**Supplementary Information:**

The online version contains supplementary material available at 10.1186/s13054-023-04775-1.

## Introduction

Invasive fungal diseases are a global threat among intensive care unit (ICU) patients [1]. Of those, *Candida* sp. is the most common pathogen involved [2] with global incidences ranging from 5.5 to 16.5 cases per 1000 ICU admissions [3–5]. The dynamics of the epidemiology of ICU-acquired candidemia (ICAC) have highlighted an increase in its incidence [6] resulting from a combination of factors including an increase in the number of patients with severe underlying disease or receiving immunosuppressors, as well as improvements in ICU supportive care, which have enabled many patients who would previously have died to survive in ICU. Beyond its incidence, the epidemiological evolution of ICU-acquired infections has also shown an increasing antifungal resistance and the emergence of *Candida* species of concern reinforcing the need for close monitoring of these infections [6–9]. The impact of these fungal infections on patients’ outcomes makes their prevention and treatment crucial [3, 10, 11].

Candida colonization, originating from the gastrointestinal tract, seems to be the first step towards severe infection [12]. Besides immunosuppression and loss of intestinal epithelial integrity, among the risk factors for ICAC, gastrointestinal colonization with *Candida* may participate to promote *Candida* bloodstream infection acquisition [13, 14]. In fact, during the 1980s, Wey et al. [15] identified Candida colonization as an independent risk factor for candidemia. Multiple-site colonization with Candida spp. is recognized as a major risk factor for invasive fungal infection in critically ill patients, and the colonization density could be a predictive value for the diagnosis of systemic candidiasis [16–18]. The death risk in patients with distinct colonized body sites is similar to patients with proven Candida invasive infection [19]. Therefore, prevention and treatment of *Candida* digestive colonization may have a significant impact on ICAC incidence.

Despite longstanding assessment of strategies to improve the diagnosis and early treatments of ICAC [20, 21], studies focusing on the prevention of these infections remain scarce. Selective digestive decontamination (SDD) was initially developed from previous animal models to prevent bacterial infections acquired in ICU using several topical antibiotics[22]. The addition of antifungal components was designed to prevent the emergence of fungal overgrowth [23], not with the primary intention of preventing acquired fungal infection. However, the impact of such a strategy on the occurrence of fungal infections has only been rarely explored in ICU patients [24–26]. Therefore, we aimed to assess the association between SDD and the rate of ICAC among mechanically ventilated ICU patients.

## Method

### Ethical considerations

The database was approved by the institutional review board (CPP SUD ESTdIRB 00009118) as well as by the National Data Protection Commission (Commission Nationale de l'Informatique et des Libertés, Number 919149). Specific information concerning this surveillance was given to all patients about the potential use of their personal data for research purposes.

### Study design and population

This study was part of the REA-REZO prospective continuous multicenter cohort surveillance. This healthcare-associated infection surveillance collects patient-level data of all adult patients hospitalized for at least 2 calendar days in any of the 220 contributing ICUs of the REA-REZO network since January 2017. Surveillance focuses on ICU-acquired infection and is discontinued when the patients either die or are discharged from ICU. The detailed protocol for data collection and monitoring is available at: https://rearezo.chu-lyon.fr/. For the present analysis, all patients hospitalized between January 2017 and January 2023 receiving mechanical ventilation for more than 48 h were included.

**Fig. 1.**
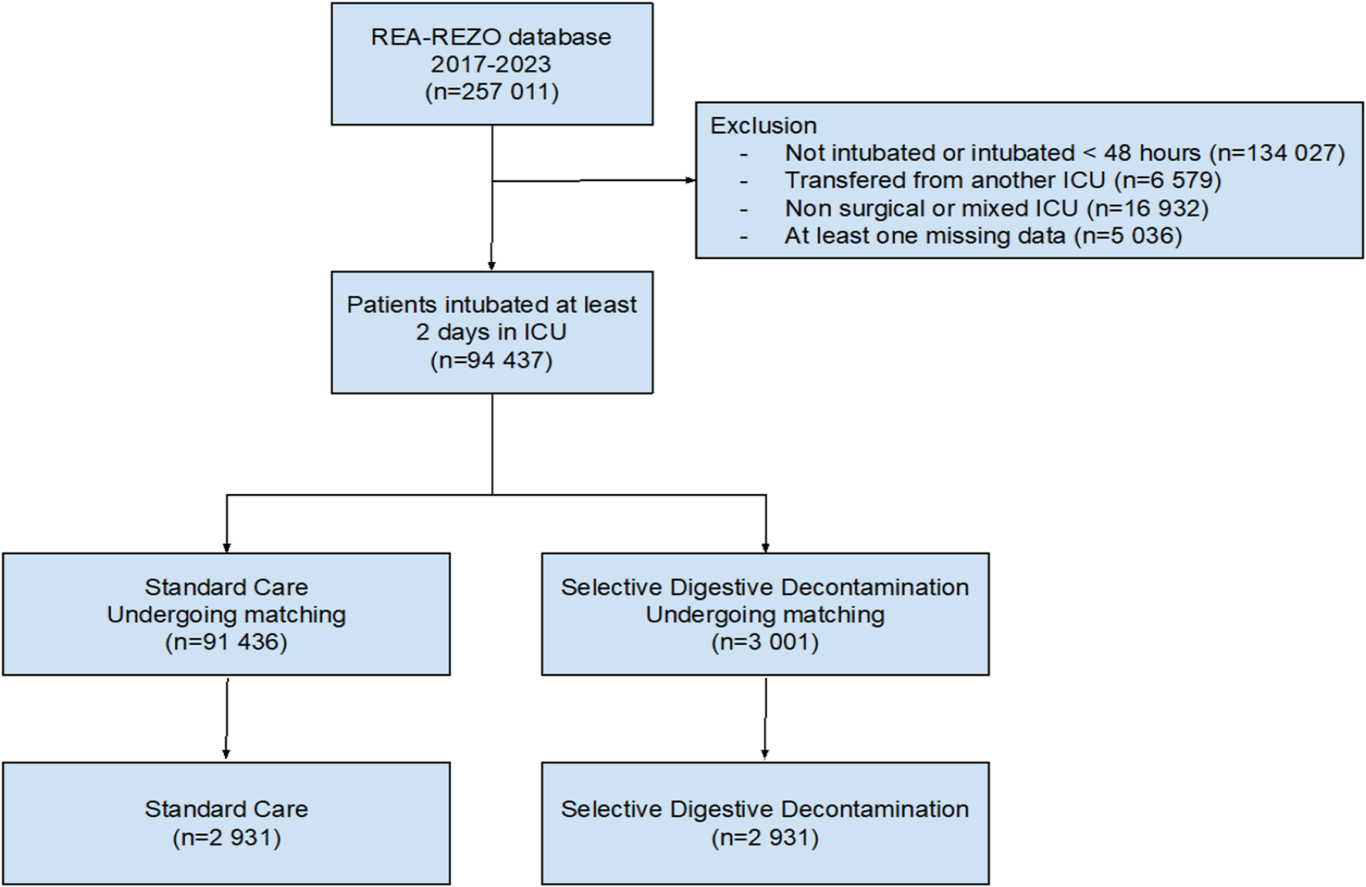
Flowchart

Patients hospitalized in other ICUs than surgical and medical-surgical ICUs were excluded since SDD was only applied in surgical and medical-surgical ICUs. Patients transferred from another ICU as well as patients with missing data were also excluded (Fig. 1).

### Intervention

Among the 220 participating intensive care units, in addition to standard care, SDD was applied systematically in 6 facilities (2.7%), while the other 214 ICUs did not use this prevention strategy. Decontamination regimens were not homogeneous across the ICUs, and details of these regimens are provided in Additional file 1: Table S1. Briefly, SDD consists of the administration of topical anti-infective drugs including aminoglycoside, colistin sulfate and amphotericin B, four times a day into the oropharynx and nasogastric tube. Detailed composition of decontamination regimens was not consistent between participating ICUs. In two participating ICUs, in addition to the oro-digestive regimen, patients were also treated with a short course of intravenous antibiotics (cefotaxime or cefazolin or fluoroquinolone in case of allergy), while in 2 others, SDD was accompanied by a daily chlorhexidine bodywash. Notably, antifungal prophylaxis was uniformly enteral amphotericin B in all six facilities. SDD was applied in intubated patients with an expected intubation duration > 24 h from admission and during full length of mechanical ventilation duration. Each center had a nosocomial infection control committee (Comité de Lutte contre les Infections Nosocomiales: CLIN) and an infection control team for the prevention and prospective census of acquired infections and applied the recommendations of the French Society for Infection Control and Prevention (available at https://sf2h.net/publications/actualisation-precautions-standard-2017). Source of candidemia was assessed at the discretion of each investigator of the REA-REZO multicenter surveillance. Systematic screening for Candida colonization was not routinely performed in the 220 participating ICUs.

### Definitions

ICAC was defined by at least one positive blood culture positive for *Candida* sp. sampled after more than 48 h of ICU stay [27]. Immunosuppression was classified as aplasia (neutrophils < 500/mm3) or other type of immunosuppression (i.e., due to treatment (chemotherapy, radiotherapy, immunosuppressants, long-term or recent high-dose corticosteroids) and/or an immunosuppressive disease (leukemia, lymphoma, AIDS) [28]. Acquisition of multi-resistant (MDR) bacteria in ICU was defined as either colonization or infection due to methicillin-resistant *Staphylococcus aureus*, glycopeptide-intermediate *Staphylococcus aureus*, glycopeptide-resistant *Enterococcus*, extended-spectrum beta-lactamase producing *Enterobacterales* (ESBLE), carbapenemase-producing *Enterobacterales*, carbapenem-resistant *Acinetobacter baumanii* or carbapenem-resistant *Pseudomonas aeruginosa*.

### Objectives

Our primary objective was to compare the rate of candidemia according to SDD of the digestive tract.

Secondary objectives included comparison of the likelihood of developing ICAC throughout the ICU stay, comparison of ICU length of stay, duration of mechanical ventilation, rates of acquisition of MDR bacteria, as well as ICU survival according to SDD.

### Statistical analysis

Data were reported as numbers (percentages) for categorical variables or medians (interquartile ranges: 25th–75th percentiles) for continuous variables. Severity was assessed by the Simplified Acute Physiological Score II [29]. To account for inter-group imbalances of baseline characteristics between SDD and standard care patients, a propensity score (PS) matched analysis with a 1:1 ratio was performed. PS corresponds for each patient to his probability to receive SDD and calculation was conducted using a non-parsimonious logistic regression model including every variable available during the period at risk for candidemia (i.e., during ICU stay in patients who did not develop candidemia and before candidemia onset for those who did). The following variables were therefore included: year of admission, season of admission, type of ICU of admission, age, sex, SAPS II, type of ICU (Surgical or Medical-Surgical), biologically confirmed COVID-19, main reason for ICU admission (secondary to a trauma or not), type of admission (medical, elective surgery or emergency surgery), provenance from community/nursing home, immunosuppression (both neutropenia and other kinds of immunosuppression), early treatment with antibiotics and use of central venous catheter before ICAC onset. Then, using the “MatchIt” package, a nearest neighbor algorithm was used for PS matching with a 1:1 ratio: each patient receiving SDD was matched with 1 patient who did not receive SDD with the nearest PS, using a caliper of 0.1. Satisfactory matching was defined as an absolute value of the standardized mean difference (SMD) < 0.1 for all variables. Continuous variables were compared using Mann Whitney and the unpaired *t* test, depending on the distribution of the data, and proportion using Chi-square tests, as appropriate. Furthermore, as age is also a component of the SAPS II score, we conducted a sensitivity analysis excluding age from the propensity score.

In addition, competing risk analysis was used to estimate the cumulative incidence of the first episodes of ICAC between study groups considering death within 90 days as a competing event in order to take into account the time-dependent nature of ICAC. Curves were compared using the Gray test, and hazard ratio (HR) with their 95% confidence interval (95% CI) was estimated using the Fine and Gray subdistribution (sd) hazard function. Proportionality assumption of the Fine and Gray model was graphically assessed over the follow-up period, and where it was not respected, follow-up time was partitioned.

Kaplan–Meier survival curves with the log-rank test were used for survival analysis.

Statistical analyses were performed with the statistical software R 4.1.1. All tests were two-sided, and *p* < 0.05 was considered statistically significant. The design of this study followed the Strengthening in Reporting of Observational Studies in Epidemiology (STROBE) guidelines [30].

## Results

### Overall population

Throughout the study period, a total of 257 011 patients were identified among the participating ICUs. Of those, we excluded 134 027 patients that were not intubated or intubated less than 48 h, 6 579 transferred from another ICU, 16 932 patients that were not hospitalized in surgical or medical-surgical ICUs and 5 036 patients who had at least one missing data. Therefore, 94 437 patients were considered for matching. SDD was administered to 3 001 patients (3.2%) (Fig. 1). The description of the full population according to SDD is displayed in Additional file 1: Table S2. Overall, a total of 651 (0.7%) patients experienced at least one episode of candidemia. The proportion of patients with ICAC was lower in the SDD group (0.3% (8/3 001) versus 0.7% (643/91 436); *p* = 0.006). Notably, the median delay of the first ICAC from ICU admission was 11 days (5–20) and did not differ according to SDD (11 days (5–21) in standard care patients versus 9 (8–12) in SDD patients; *p* = 0.660). Furthermore, patients with ICAC had higher ICU mortality rate as compared with those that did not develop ICAC (48.4% versus 29.8%; *p* < 0.001). *Candida albicans* was the most common *Candida* species recovered from blood cultures accounting for 60.4% of all *Candida* species (Additional file 1: Table S3).

### Propensity score matched analysis

**Fig. 2.**
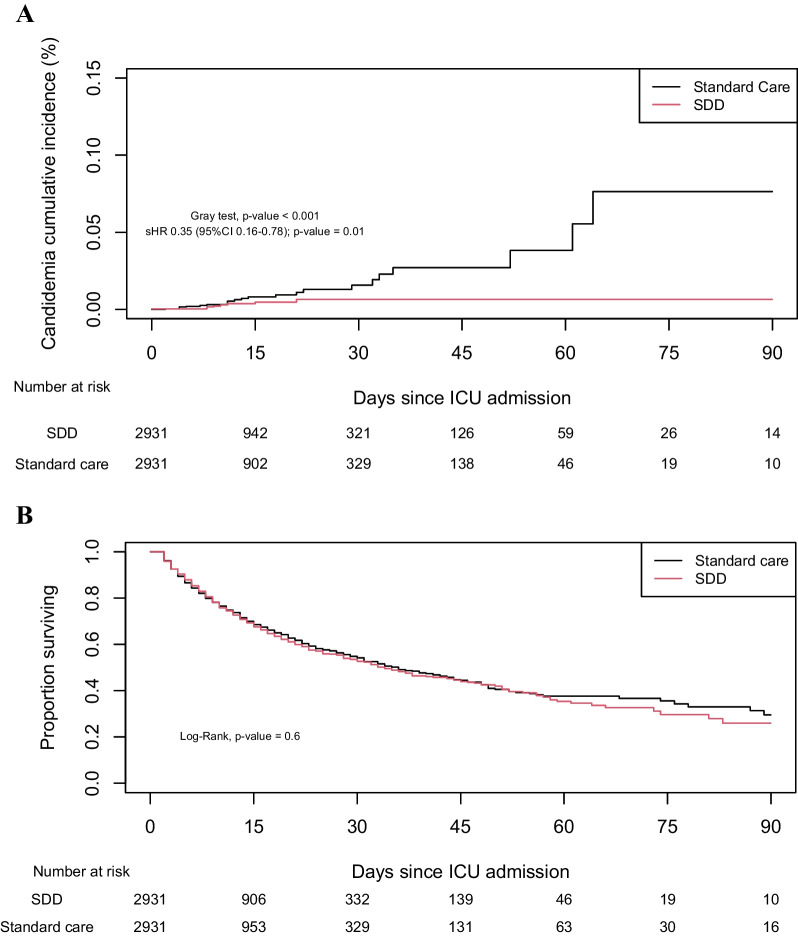
Cumulative incidence of candidemia (**A**) and survival analysis (**B**) according to selective digestive decontamination or standard care in the matched population

In order to overcome baseline differences between groups, a propensity score matched analysis was performed. The density plot of the propensity score of included patients is displayed in Additional file 2: Figure S1. The baseline characteristics between the two groups were reassessed after propensity score matching. The standardized mean differences of each variable are shown in Additional file 2: Figure S2. The propensity score matching included 2 931 patients in the SDD group and in the standard care group. The baseline characteristics between the two groups after propensity score matching were well balanced (SMD < 0.1) (Table 1). In the matched population, the proportion of patients developing ICAC was lower in the SDD group as compared to the standard care group (0.3% versus 0.8%; *p* = 0.012) as presented in Fig. 2A and in Table 2. Cumulative incidence analysis also showed a decreased incidence of ICAC in SDD patients (Gray test *p* < 0.001). Furthermore, when performing competing risk analysis, such an association between SDD and decreased rate of ICAC was also observed (sdHR = 0.35 [95% CI 0.16–0.78]; *p* = 0.01). The rate of ICU-acquired MDR bacteria was lower in SDD patients compared to patients receiving standard care (1.2% versus 4.6%; *p* < 0.001). The proportionality of hazard was not respected and the risk of ICAC significantly decreased after day 10 following ICU admission in patients receiving SDD. After introducing a time-dependent variable, we estimated a sHR for SDD patients of 0.19 (95% CI 0.05–0.65; *p* = 0.008) after Day 10 following ICU admission. In addition, duration of mechanical ventilation and ICU length of stay did not differ between the two groups of patients (6 days (3–12) versus 6 days (3–12); *p* = 0.120 and 9 days (5–18) versus 9 days (5–17); *p* = 0.513). Finally, mortality analysis did not show any difference between groups (Fig. 2B). Of note, among patients in the standard care group who were not matched, the ICAC rate was 0.7% (620/88 505).

**Table 1:** Characteristics of matched patients whether or not they received selective digestive decontamination

	All patients n = 5862	Standard care n = 2931	SDD n = 2931	SMD
*Year of ICU admission*				
2017	895 (15.3)	444 (15.1)	451 (15.4)	0.0067
2018	817 (13.9)	407 (13.9)	410 (14.0)	0.0030
2019	652 (11.1)	313 (10.7)	339 (11.6)	0.0280
2020	460 (7.8)	220 (7.5)	240 (8.2)	0.0252
2021	1092 (18.6)	560 (19.1)	532 (18.2)	−0.0250
2022	1946 (33.2)	987 (33.7)	959 (32.7)	−0.0201
*Season of ICU admission*				
Spring	1397 (23.8)	711 (24.3)	686 (23.4)	−0.0203
Summer	1446 (24.7)	690 (23.5)	756 (25.8)	0.0513
Fall	1678 (28.6)	850 (29.0)	828 (28.2)	−0.0166
Winter	1341 (22.9)	680 (23.2)	661 (22.6)	−0.0156
Type of ICU				
Medical-surgical (vs surgical)	5118 (87.3)	2538 (86.6)	2580 (88.0)	0.0446
*Baseline characteristics*				
Age (years)	63 [50–72]	63 [50–73]	63 [50–72]	−0.0292
Male sex	4024 (68.7)	2003 (68.3)	2021 (69.0)	0.0133
Immunosuppression				
No immunodepression	5228 (89.2)	2614 (89.2)	2614 (89.2)	0.0000
Neutropenia	249 (4.2)	122 (4.2)	127 (4.3)	0.0077
Other immunosuppression	385 (6.6)	195 (6.7)	190 (6.5)	−0.0070
Simplified acute physiology score II	54 [40–68]	55 [41–68]	54 [40–68]	−0.0547
Reason for ICU admission: Trauma	1523 (26.0)	764 (26.1)	759 (25.9)	−0.0038
*Type of admission*				
Medical	3417 (58.3)	1720 (58.7)	1697 (57.9)	−0.0158
Elective surgery	341 (5.8)	168 (5.7)	173 (5.9)	0.0073
Emergency surgery	2104 (35.9)	1043 (35.6)	1061 (36.2)	0.0127
COVID 19	468 (8.0)	237 (8.1)	231 (7.9)	−0.0077
Provenance from community or nursing home	3941 (67.2)	1959 (66.8)	1982 (67.6)	0.0169
*Clinical course*				
Antibiotherapy at admission	2946 (50.3)	1488 (50.8)	1458 (49.7)	−0.0205
Central venous catheter	5162 (88.1)	2587 (88.3)	2575 (87.9)	−0.0126

Data are presented as median [IQR: interquartiles], n (%)

*COVID-19* Coronavirus disease 2019, *HAS* hydroalcoholic solution, *ICU* intensive care unit, *SDD* selective digestive decontamination, *SMD* standardized mean difference

**Table 2:** Main outcomes of matched patients whether or not they received selective digestive decontamination

	All patients n = 5862	Standard care n = 2931	SDD n = 2931	*p *value
*Ouctomes*				
Candidemia	31 (0.5)	23 (0.8)	8 (0.3)	0.012
Sources of candidemia^a^				0.65
Catheter	9 (29.0)	7 (30.4)	2 (25.0)	
Digestive	6 (19.3)	5 (21.7)	1 (12.5)	
Other or unknown	14 (45.2)	10 (43.5)	4 (50.0)	
Pleuro-pulmonary site	1 (3.2)	1 (4.3)	0 (0.0)	
Skin	1 (3.2)	0 (0.0)	1 (12.5)	
*Candida* species isolated				0.186
*Candida albicans*	21 (67.7)	16 (69.6)	5 (62.5)	
*Candida glabrata*	3 (9.7)	3 (13.0)	0 (0.0)	
*Candida parapsilosis*	1 (3.2)	1 (4.3)	0 (0.0)	
*Candida tropicalis*	2 (6.4)	2 (8.7)	0 (0.0)	
*Candida krusei*	2 (6.4)	1 (4.3)	1 (12.5)	
Other *Candida* species	2 (6.4)	0 (0.0)	2 (25.0)	
MDR bacteria acquisition^b^	103 (2.9)	82 (4.6)	21 (1.2)	<0.001
ICU length of stay (days)	9 [5–18]	9 [5–18]	9 [5–17]	0.513
Duration of mechanical ventilation (days)	6 [3–12]	6 [3–12]	6 [3–21]	0.120
ICU case fatality	1821 (31.1)	908 (31.0)	913 (31.1)	0.910

Data are presented as median (IQR: interquartiles), n (%)

*ICU* intensive care unit, *SDD*: selective digestive decontamination, *MDR* multidrug resistant

^a^Source of candidemia was assessed when colonization with the same Candida Spp was identified as causative pathogen

^b^Missing data: n = 2364

### Sensitivity analysis

When excluding the patient's age from propensity score development, balanced populations were also obtained (Additional file 1: Table S4). Assessment of the ICAC rate in this matched population revealed a similar association between SDD and reduced ICAC rate, with 0.3% of patients developing ICAC in SDD patients versus 0.8% in patients who did not receive SDD (*p* = 0.005) (Additional file 1: Table S5).

## Discussion

In the present large cohort study including ICU patients receiving mechanical ventilation for at least 48 h, we observed a significant reduction in ICAC among those receiving SDD.

Improvements in the management of ICU patients over the past decades have unmasked the impact of secondary infections [31] making the prevention of such infections crucial for clinicians. Of those infections, *Candida* species are the main fungal pathogens involved. While strategies to prevent ICU-acquired infections have mainly focused on bacterial sepsis, the prevention of fungal infections remains under-investigated. One explanation could be the low incidence of these infections, compared to bacterial sepsis. Our data show the proportion of patients developing candidemia is 0.7% in the overall population, which is close to what has been reported in other previous studies [3, 27].

Such a low rate makes it difficult to design randomized clinical trials, as a very large cohort of patients would be needed to achieve sufficient power for such therapeutic trials. Therefore, the use of registries, by including large cohorts of patients, makes it possible to overcome these methodological issues when studying low-incidence diseases.

In our study, the impact of ICAC on the fate of patients, with a survival rate dropping in the overall population from 70.2 to 51.6%, deserves to be highlighted. Therefore, although having a low incidence rate (compared to other ICU-acquired infections), the consequences of ICAC make their prevention a priority [11].

Our results on the effect of SDD on ICAC are consistent with previous studies reporting very low incidence of candidemia in ICU patients receiving SDD suggesting another positive effect of SDD in addition to reducing VAP and bacteremia and improving ICU patient outcomes [22, 24–26]. Although recommended in ICUs where the prevalence of MDR bacteria is low (< 20%) as a validated strategy to prevent VAP in recent guidelines [32], implementation of SDD in ICUs remains low [33]. In the present study, only 3.2% of the patients included benefited from such a preventive strategy which may limit the generalizability of the findings. Moreover, the low proportion of ICUs applying SDD might drive remaining residual confounders such as other measures to prevent nosocomial infections including candidaemia. Factors that may have contributed to the low compliance with current guidelines may include the resources required to implement such a strategy. However, the use of resources can be offset by the reduction in the duration of mechanical ventilation and the decreased rate of healthcare-associated infections observed in previous studies [22, 34]. In addition, fear of antimicrobial resistance may prevent clinicians from implementing SDD. Nonetheless, studies deciphering this issue evidenced the absence of effect of SDD regimens on multidrug-resistant bacteria colonization and acquired infections [35, 36]. Moreover, in the present analysis, the rate of MDR bacteria acquisition in ICU appeared lower in SDD patients. Beyond multidrug-resistant bacteria, a global concern is the emergence of antifungal-resistant yeast. The growing incidence of azole and echinocandin resistances represents major challenges for therapeutic strategies [37]. While previous studies assessed the impact of SDD on antibiotics resistance, to the best of our knowledge, the effect of administering amphotericin B to patients receiving SDD on antifungal resistance remains unexplored. Moreover, recent outbreaks of *Candida auris* infections could change the fungal landscape of ICU patients. Since exposure to fluconazole is a predictive factor for these multi-resistant yeasts infections [8], preventing ICAC could help limit the spread of these threatening pathogens. Neither resistance to antifungal agents nor colonization by *Candida* has been assessed in our study, leaving this question unanswered.

The reduction of ICAC might be explained by several reasons including lower incidence of *Candida* digestive colonization promoted by antifungal components of SDD. Notably, our study reveals that the effects of SDD on ICAC seemed to appear particularly in those whose source was digestive suggesting that the prevention of ICAC might be promoted by decreased digestive Candida colonization especially in patients at risk for intra-abdominal candidiasis. Similarly, a previous study evidenced a substantial effect of SDD in surgical patients [38]. Furthermore, by potentially reducing healthcare-associated infections, SDD could reduce the need for broad-spectrum antibiotic therapy and ICU stay which are risk factors for invasive candidiasis[39, 40]. Noteworthy, our results suggest that the impact of SDD on ICAC appears significant after the 10th day following ICU admission. Although the majority of patients stay in ICU for less than 30 days, we did not observe an early effect of SDD on ICAC, whereas the long-term effect was more pronounced. Such an observation suggests that SDD may only be beneficial in patients with longer ICU length of stay. There may be several reasons for this long-term effect including the timing of ICAC occurring at a median delay of 10 days after ICU admission (IQR 5–20), thus precluding the observation of an early effect. In addition, it may be supposed that SDD, by preventing early bacterial ICU-acquired infections, could result in a reduction of sepsis-associated immunoparalysis [41] that could favor late acquisition of invasive fungal infection [42].

In the present study, we did not observe any SDD-related benefit on patients’ survival. However, our work was not designed to assess this question and many confounding factors may be involved. Furthermore, given the low rate of ICAC in our study population, the benefit of SDD on ICAC could not translate into a statistically significant lower mortality rate (0.6% of the matched population developing ICAC as compared to the 30.6% mortality rate). Along these lines, a recent large-scale randomized clinical trial in mechanically ventilated patients also did not evidenced any effect of SDD on in-hospital mortality[43]. However, the results of this trial suggested a clinically important benefit. In addition, a meta-analysis including this trial was published simultaneously showing lower in-hospital mortality for patients treated with SDD [34].

Our study is, to our knowledge, the largest one to explore the effects of SDD on ICAC. However, some limitations must be acknowledged. Firstly, while our findings align with previous cohort studies, it is important to note that residual confounding factors are inherent to the observational nature of our study and may have been exacerbated by limitations in data availability (such as important predictors of nosocomial transmission, namely infection control, early use of antifungals, abdominal surgery, parenteral nutrition, acute kidney injury requiring renal replacement therapy, etc.), thus limiting the ability to draw definitive conclusions. A randomized controlled trial would be needed to conclude. Furthermore, given that facilities treating their patients with SDD use it for every intubated patient, we were unable to account for a possible center effect contributing to a possible residual bias. Secondly, we did not evaluate the effects of SDD on potential Candida cross-transmission between patients [23], given that in each institution treating its patients with SDD, all mechanically ventilated patients are treated with SDD. Thirdly, the follow-up of the included patients was restricted to their stay in the ICU. Therefore, the long-term effects of SDD on patients' outcomes, especially the potential rebound of invasive fungal infections upon withdrawal of SDD, could not be assessed. Nonetheless, SDD being stopped at the end of mechanical ventilation (*i.e.,* before ICU discharge), such a rebound would have been observed in the present analysis. In addition, the effects of SDD on exposure to antibiotics or antifungals could not be assessed due to the limited availability of data. While previous studies showed a protective effect of SDD on antibiotic resistance, to the best of our knowledge, the effect of administering amphotericin to patients receiving SDD on antifungal resistance remains unexplored. Although challenging, the assessment of individual and environmental long-term ecological impacts of SDD deserves to be investigated. Furthermore, some Candida species, such as *Candida lusitaniae* or *Candida Haemulonii* can be resistant to amphotericin B, making antifungal components of SDD possibly ineffective against these strains [44, 45]. Fourthly, the low number of ICUs using SDD causing an imbalance in the design can introduce some bias in the results. However, the protective effect of SDD assessed in the present study could help to convince French ICU physicians to use such a strategy. In addition, among the SDD group, different SDD protocols were used across ICUs that may have caused heterogeneous effects on ICAC as well as on patients' survival. Nonetheless, despite different antibacterial regimens, the use of enteral amphotericin B was similar between all the ICUs of the SDD group. Finally, despite a lower ICAC rate, SDD patients had a similar ICU mortality rate which may be explained by the marginal effect of ICAC in our study population where the incidence of ICAC is low as compared to the overall mortality rate.

In conclusion, in this study with low prevalence of ICAC, SDD was associated with a lower rate of ICAC that did not translate to higher survival.

### Supplementary Information

**Additional file 1.** Supplementary Tables 1–5.

**Additional file 2.** Supplementary Figures 1–2.

## Data Availability

The datasets from this study are available from the corresponding author on request.
