# Supplementary material for: Association between selective digestive decontamination and decreased rate of acquired candidemia in mechanically ventilated ICU patients: a multicenter nationwide study
Source: Crit Care. 2023 Dec 16;27:494. doi: 10.1186/s13054-023-04775-1 (PMC10724923; doi:10.1186/s13054-023-04775-1)
Supplement: Supplementary file 1 — Additional file 1. Supplementary Tables 1–5. [file 13054_2023_4775_MOESM1_ESM.docx]

**Supplementary Table 1.** Characteristics of the selective digestive decontamination treatments in the different ICUs

| **Hospital centers** | **Components of SDD** | **Frequency** | **Patients receiving SDD** |
| --- | --- | --- | --- |
| ICU of the Vannes hospital center | Gentamycin 133 mg  Enteral amphotericin B 500mg Colistin 100mG | QID | Patients receiving mechanical ventilation |
| ICU of the Annecy-Genevois hospital center | Gentamycin 133 mg  Enteral amphotericin B 500mg Colistin 100mG  Intravenous Cefotaxime 1G QID during 4 days | QID | Patients receiving mechanical ventilation during at least 48 hours |
| ICU of the Saint-Brieuc hospital center | Gentamycin 133 mg  Enteral amphotericin B 500mg Colistin 100mG | QID | Patients receiving mechanical ventilation |
| ICU of the Quimper hospital center | Tobramycin 80 mg  Enteral amphotericin B 500mg Colistin 100mG | QID | Patients receiving mechanical ventilation |
| Surgical ICU of the Nimes academic hospital center | Tobramycin 80 mg  Enteral amphotericin B 500mg Colistin 100mG  Intravenous Cefazolin 2G TID during 1 day | QID | Patients receiving mechanical ventilation |
| Surgical ICU of the Marseille Nord academic hospital center | Tobramycin 80 mg  Enteral amphotericin B 500mg Colistin 100mG  Intravenous Cefazolin 2G TID during 2 days | QID | Patients receiving mechanical ventilation |

Abbreviations: QID: *Quater in die*; TID: *Ter in die*

**Supplementary Table 2.** Characteristics of patients whether or not they received selective digestive decontamination

|  | **All patients**  **n=94437** | **Standard care**  **n=91436** | **SDD**  **n=3001** | ***p* value** |
| --- | --- | --- | --- | --- |
| Year of ICU admission |  |  |  | <0.001 |
| 2017 | 30996 (32.8) | 30545 (33.4) | 451 (15.0) |  |
| 2018 | 21388 (26.6) | 20978 (22.9) | 410 (13.7) |  |
| 2019 | 12232 (12.9) | 11893 (13.0) | 339 (11.3) |  |
| 2020 | 10503 (11.1) | 10263 (11.2) | 240 (8.0) |  |
| 2021 | 10585 (11.2) | 10051 (11.0) | 534 (17.8) |  |
| 2022 | 8733 (9.2) | 7706 (8.4) | 1027 (34.2) |  |
| Season of ICU admission |  |  |  | <0.001 |
| Spring | 23960 (25.4) | 23268 (25.4) | 692 (23.1) |  |
| Summer | 21631 (22.9) | 20849 (22.8) | 782 (26.1) |  |
| Fall | 23088 (24.4) | 22233 (24.3) | 855 (28.5) |  |
| Winter | 25758 (27.3) | 25086 (27.4) | 672 (22.4) |  |
| Type of ICU |  |  |  |  |
| Medical-Surgical | 81358 (86.1) | 78708 (86.1) | 2650 (88.3) | 0.001 |
| **Baseline characteristics** |  |  |  |  |
| Age (years) | 67 (56-75) | 67 [56-75] | 63 [50-72] | <0.001 |
| Male sex | 61700 (65.3) | 59622 (65.2) | 2078 (69.2) | <0.001 |
| Immunosuppression |  |  |  | <0.001 |
| No immunodepression | 81625 (86.4) | 78971 (86.4) | 2654 (88.4) |  |
| Neutropenia | 1447 (1.5) | 1290 (1.4) | 157 (5.2) |  |
| Other immunosuppression | 11365 (12.0) | 11175 (12.2) | 190 (6.3) |  |
| Simplified acute physiology score II | 52 [39-66] | 52 [39-66] | 54 [40-68] | 0.005 |
| Reason for ICU admission: Trauma | 7700 (8.1) | 6875 (7.5) | 825 (27.5) | <0.001 |
| Type of admission |  |  |  | <0.001 |
| Medical | 65476 (69.3) | 63773 (69.7) | 1703 (56.7) |  |
| Elective surgery | 8487 (9.0) | 8314 (9.1) | 173 (5.8) |  |
| Emergency surgery | 20474 (21.7) | 19349 (21.2) | 1125 (37.5) |  |
| COVID 19 | 5884 (6.2) | 5652 (6.2) | 232 (7.7) | <0.001 |
| Provenance from community or nursing home | 56031 (59.3) | 53980 (59.0) | 2051 (68.3) | <0.001 |
| **Clinical course** |  |  |  |  |
| Antibiotherapy at admission | 60038 (63.6) | 58553 (64.0) | 1485 (49.5) | <0.001 |
| Central venous catheter | 77928 (82.5) | 75285 (82.3) | 2643 (88.1) | <0.001 |

Data are presented as median [IQR: interquartiles], n (%).

Abbreviations: COVID-19: Coronavirus disease 2019; HAS: Hydroalcoholic solution; ICU: Intensive Care Unit; SDD: Selective Digestive Decontamination,

**Supplementary Table 3.** Comparisons of main outcomes of patients whether or not they received selective digestive decontamination.

|  | **All patients**  **n=94437** | **Standard care**  **n=91436** | **SDD**  **n=3001** | ***p-value*** |
| --- | --- | --- | --- | --- |
| **Ouctomes** |  |  |  |  |
| Candidaemia | 651 (0.7) | 643 (0.7) | 8 (0.3) | 0.006 |
| Sources of candidaemia (n=651) ^a^ |  |  |  | 0.343 |
| Catheter | 155 (23.8) | 153 (23.8) | 2 (25.0) |  |
| Digestive tract | 143 (22.0) | 142 (22.1) | 1 (12.5) |  |
| Other or unknown | 255 (39.2) | 251 (37.5) | 4 (50.0) |  |
| Pleuro-pulmonary site | 58 (8.9) | 58 (9.0) | 0 (0.0) |  |
| Urinary tract | 30 (4.6) | 30 (4.7) | 0 (0.0) |  |
| Skin | 10 (1.5) | 9 (1.4) | 1 (12.5) |  |
| *Candida* species isolated (n=651) |  |  |  | 0.112 |
| *Candida albicans* | 393 (60.4) | 388 (60.3) | 5 (62.5) |  |
| *Candida glabrata* | 95 (14.6) | 95 (14.8) | 0 (0.0) |  |
| *Candida parapsilosis* | 73 (11.2) | 73 (11.3) | 0 (0.0) |  |
| *Candida tropicalis* | 26 (4.0) | 26 (4.0) | 0 (0.0) |  |
| *Candida krusei* | 15 (2.3) | 14 (2.2) | 1 (12.5) |  |
| Other *Candida* species | 49 (7.5) | 47 (7.3) | 2 (25.0) |  |
| MDR bacteria acquisition ^b^ | 861 (1.1) | 853 (1.1) | 8 (0.4) | <0.001 |
| ICU length of stay (days) | 9 [5-17] | 9 [5-17] | 9 [5-17] | 0.464 |
| Duration of mechanical ventilation (days) | 6 [3-12] | 6 [3-12] | 6 [3-12] | 0.384 |
| ICU case fatality | 28248 (29.9) | 27319 (29.9) | 929 (31.0) | 0.211 |

Data are presented as median (IQR: interquartile), n (%).

Abbreviations: ICU: Intensive Care Unit; CI: Confidence Interval; SDD: Selective Digestive Decontamination, MDR: multidrug resistant

^a^ Source of candidemia was assessed when colonization with the same Candida Spp was identified as causative pathogen.

^b^ Missing data: n= 64618

**Supplementary Table 4.** Characteristics of matched patients whether or not they received selective digestive decontamination (matching process that did not include patient’s age)

|  | **All patients**  **n=5886** | **Standard care**  **n=2943** | **SDD**  **n=2943** | **SMD** |
| --- | --- | --- | --- | --- |
| Year of ICU admission |  |  |  |  |
| 2017 | 908 (15.4) | 457 (15.5) | 451 (15.3) | -0.0057 |
| 2018 | 819 (13.9) | 409 (13.9) | 410 (13.9) | -0.0198 |
| 2019 | 595 (10.1) | 256 (8.7) | 339 (11.5) | 0.0568 |
| 2020 | 539 (9.2) | 299 (10.2) | 240 (8.2) | 0.0801 |
| 2021 | 1118 (19.0) | 588 (20.0) | 530 (18.0) | -0.0408 |
| 2022 | 1907 (32.4) | 934 (31.7) | 973 (33.1) | -0.0322 |
| Season of ICU admission |  |  |  |  |
| Spring | 1342 (22.8) | 655 (22.3) | 687 (23.3) | -0.0185 |
| Summer | 1531(26.0) | 770 (26.2) | 761 (25.9) | 0.0093 |
| Fall | 1679 (28.5) | 851 (28.9) | 828 (28.1) | 0.0263 |
| Winter | 1334 (22.7) | 667 (22.7) | 667 (22.7) | 0.0093 |
| Type of ICU |  |  |  |  |
| Medical-Surgical (vs surgical) | 5156 (87.6) | 2564 (87.1) | 2592 (88.1) | -0.0137 |
| **Baseline characteristics** |  |  |  |  |
| Male sex | 4089 (69.5) | 2054 (69.8) | 2035 (69.1) | -0.0066 |
| Immunosuppression |  |  |  |  |
| No immunodepression | 5292 (89.9) | 2666 (90.6) | 2626 (89.2) | -0.0467 |
| Neutropenia | 229 (3.9) | 102 (3.5) | 127 (4.3) | 0.0396 |
| Other immunosuppression | 365 (6.2) | 175 (5.9) | 190 (6.5) | 0.0251 |
| Simplified acute physiology score II | 53 [40-67] | 53.00 [40.00, 67.00] | 54.00 [40.00, 68.00] | 0.0417 |
| Reason for ICU admission: Trauma | 1563 (26.5) | 795 (27.0) | 768 (26.1) | -0.0084 |
| Type of admission |  |  |  |  |
| Medical | 3415 (58.0) | 1717 (58.3) | 1698 (57.7) | -0.0445 |
| Elective surgery | 331 (5.6) | 158 (5.4) | 173 (5.9) | 0.0277 |
| Emergency surgery | 2140 (36.3) | 1068 (36.3) | 1072 (36.4) | 0.0322 |
| COVID 19 | 460 (7.8) | 229 (10.0) | 231 (10.4) | -0.0534 |
| Provenance from community or nursing home | 4000 (68.0) | 2005 (68.1) | 1995 (67.8) | 0.0423 |
| **Clinical course** |  |  |  |  |
| Antibiotherapy at admission | 2936 (49.9) | 1478 (50.2) | 1458 (49.5) | -0.0224 |
| Central venous catheter | 5180 (88.0) | 2592 (88.1) | 2588 (87.9) | -0.0283 |

Data are presented as median [IQR: interquartiles], n (%).

Abbreviations: COVID-19: Coronavirus disease 2019; HAS: Hydroalcoholic solution; ICU: Intensive Care Unit; SDD: Selective Digestive Decontamination, SMD: Standardized Mean Difference

**Supplementary Table 5.** Main outcomes of matched patients whether or not they received selective digestive decontamination (matching process that did not include patient’s age)

|  | **All patients**  **n=5886** | **Standard care**  **n=2943** | **SDD**  **n=2943** | ***p-*value** |
| --- | --- | --- | --- | --- |
| **Ouctomes** |  |  |  |  |
| Candidaemia | 33 (0.6) | 25 (0.8) | 8 (0.3) | 0.005 |
| Sources of candidaemia^a^ |  |  |  | 0.460 |
| Catheter | 9 (27.3) | 7 (28.0) | 2 (25.0) |  |
| Digestive | 7 (21.2) | 6 (24.0) | 1 (12.5) |  |
| Other or unknown | 13 (39.4) | 9 (36.0) | 4 (50.0) |  |
| Pleuro-pulmonary site | 3 (9.1) | 3 (12.0) | 0 (0.0) |  |
| Skin | 1 (3.0) | 0 (0.0) | 1 (12.5) |  |
| *Candida* species isolated |  |  |  | 0.945 |
| *Candida albicans* | 19 (57.6) | 14 (56.0) | 5 (62.5) |  |
| *Candida glabrata* | 6 (6.1) | 2 (8.0) | 0 (0.0) |  |
| *Candida parapsilosis* | 1 (3.0) | 1 (4.0) | 0 (0.0) |  |
| *Candida tropicalis* | 1 (3.0) | 1 (4.0) | 0 (0.0) |  |
| *Candida krusei* | 2 (6.0) | 1 (4.0) | 1 (12.5) |  |
| Other *Candida* species | 8 (24.2) | 6 (24.0) | 2 (25.0) |  |
| MDR bacteria acquisition^b^ | 132 (3.7) | 111 (3.8) | 21 (0.7) | <0.001 |
| ICU length of stay (days) | 9 [5-18] | 10 [5-9] | 9 [5-17] | 0.002 |
| Duration of mechanical ventilation (days) | 6 [3-12] | 6 [3-13] | 6 [3-12] | <0.001 |
| ICU case fatality | 1804 (30.6) | 891 (30.3) | 913 (31.0) | 0.553 |

Data are presented as median (IQR: interquartiles), n (%).

Abbreviations: ICU: Intensive Care Unit SDD: Selective Digestive Decontamination, MDR: multidrug resistant

^a^ Source of candidemia was assessed when colonization with the same Candida Spp was identified as causative pathogen.

^b^ Missing data: n= 2322
