# Supplementary material for: Association between selective digestive decontamination and decreased rate of acquired candidemia in mechanically ventilated ICU patients: a multicenter nationwide study
Source: Crit Care. 2023 Dec 16;27:494. doi: 10.1186/s13054-023-04775-1 (PMC10724923; doi:10.1186/s13054-023-04775-1)
Supplement: Supplementary file 2 — Additional file 2. Supplementary Figures 1–2. [file 13054_2023_4775_MOESM2_ESM.pptx]

## Slide 1
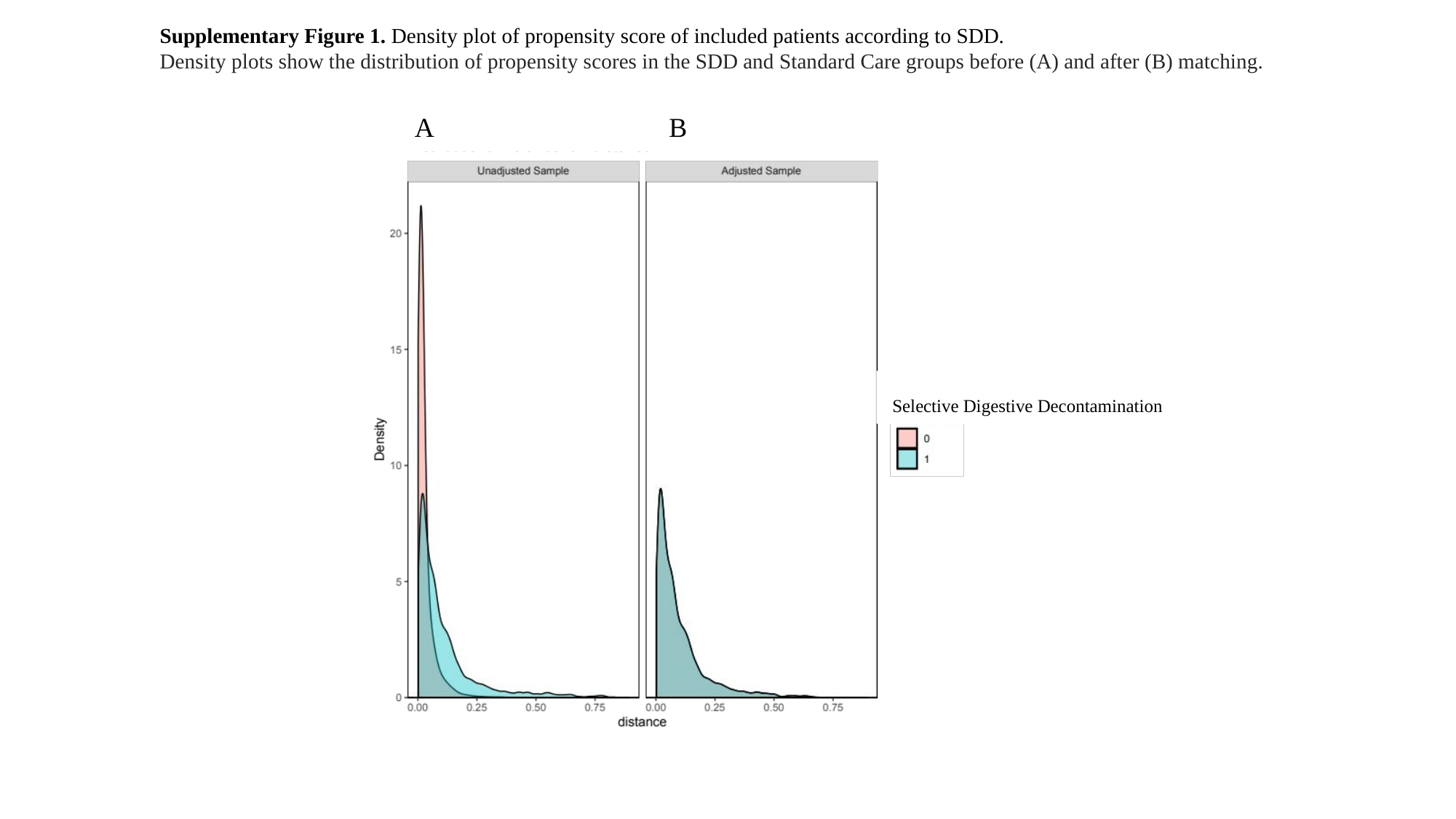

Supplementary Figure 1. Density plot of propensity score of included patients according to SDD.
Density plots show the distribution of propensity scores in the SDD and Standard Care groups before (A) and after (B) matching.
A
B
Selective Digestive Decontamination

## Slide 2
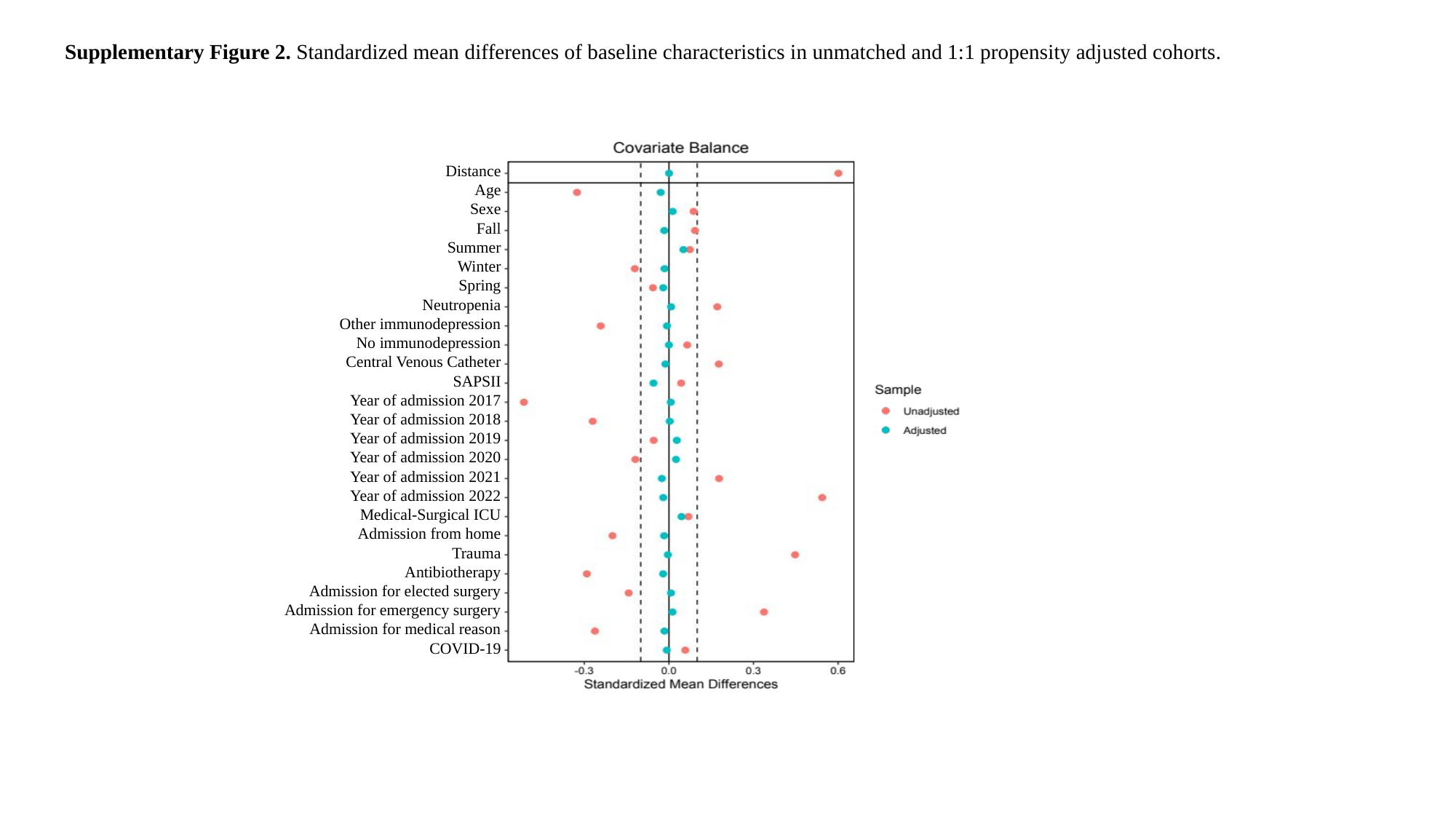

Supplementary Figure 2. Standardized mean differences of baseline characteristics in unmatched and 1:1 propensity adjusted cohorts.
Distance
Age
Sexe
Fall
Summer
Winter
Spring
Neutropenia
Other immunodepression
No immunodepression
Central Venous Catheter
SAPSII
Year of admission 2017
Year of admission 2018
Year of admission 2019
Year of admission 2020
Year of admission 2021
Year of admission 2022
Medical-Surgical ICU
Admission from home
Trauma
Antibiotherapy
Admission for elected surgery
Admission for emergency surgery
Admission for medical reason
COVID-19
